# The Effects of an Online Yoga Nidra Meditation on Subjective Well‐Being and Diurnal Salivary Cortisol: A Randomised Controlled Trial

**DOI:** 10.1002/smi.70049

**Published:** 2025-05-15

**Authors:** Esther N. Moszeik, Nicolas Rohleder, Karl‐Heinz Renner

**Affiliations:** ^1^ University of the Bundeswehr Munich Neubiberg Germany; ^2^ Chair of Health Psychology Erlangen Germany

**Keywords:** meditation, mindfulness, stress management, well‐being, yoga nidra

## Abstract

Yoga Nidra meditation has been increasingly examined in recent years for its potential to enhance psychological well‐being. However, few studies have examined its biological effects—such as diurnal cortisol patterns particularly in larger samples using pre‐post designs. The primary objective of this randomised controlled trial was to examine both the psychological (stress, anxiety, depression, rumination, sleep, satisfaction with life) and the biological effects (diurnal salivary cortisol) of Yoga Nidra. Participants were randomly assigned to 1 of 2 intervention groups (EG1: 11 min Yoga Nidra, *n* = 101; EG2: 30 min Yoga Nidra, *n* = 80), an active control group (AC: 10 min music, *n* = 74), or a waitlist control group (WC, *n* = 107). The intervention was delivered online via pre‐recorded audio files and practiced ideally daily over 2 months. Significant improvements were observed for the 11‐min Yoga Nidra group compared to the WC (effect sizes *d* = 0.08–0.16). Regular practice was associated with reductions in total cortisol and steeper diurnal slopes. Additionally, the short form significantly reduced depression compared to the AC (*d =* 0.13). The long form of Yoga Nidra showed an increase in acting with awareness (*d* = 0.10) compared to the short form. It also exceeded the effects of EG1 when compared to the AC and WC, including a flatter cortisol wake‐up reaction. The importance of small effects through economic interventions for health‐promoting behaviour is highlighted.

## 1 Introduction

‘Staying healthy means being able to relax’ is the headline of a current recommendation by the Bayerisches Staatsministerium für Gesundheit, Pflege und Prävention (Bavarian State Ministry for Health, Care and Prevention, 2022) and highlights the need to manage stress effectively. Managing stress effectively has become increasingly important, especially since the COVID‐19 pandemic (Bujard et al. 2021). Meditation is widely recognised as a method to promote mental and physical balance, with approximately 20% of the German population engaging in or considering meditation (Cramer, 2019; as cited in Esch 2021). One specific form, Yoga Nidra, has gained attention for its effects on psychological well‐being (Wahbeh and Nelson 2019) and more recently for its potential physiological benefits, including its influence on cortisol levels (Borchardt et al. 2012) or Covid‐19 symptoms (Bhardwaj et al. 2022). Yoga Nidra, or ‘yogic sleep,’ is a structured meditation practice performed in a lying position. It guides practitioners through progressive relaxation and awareness stages, facilitating deep rest (Kjaer et al. 2002). Recent research suggests that Yoga Nidra can support emotional regulation, stress reduction, and sleep improvement (Sharpe et al. 2021). However, while many studies highlight psychological benefits, fewer have rigorously examined biological stress markers such as diurnal cortisol patterns, particularly in randomised controlled trials with pre‐post designs. Cortisol, a key biomarker of the Hypothalamic‐Pituitary‐Adrenal (HPA) axis, plays a crucial role in stress regulation. Deviations in cortisol awakening response (CAR) and diurnal flattening have been linked to chronic stress and mental health issues (G. E. Miller et al. 2007; Pretscher et al. 2021). While mindfulness interventions have shown some effects on cortisol regulation, findings remain inconsistent (Engert et al. 2018). More robust, controlled studies are needed to determine whether Yoga Nidra influences both psychological and biological stress regulation. Furthermore, current studies emphasise the need for a comprehensive theoretical framework, experienced trainers, and the inclusion of active control groups (cf. Lao et al. 2016). Previous studies on the effectiveness of mindfulness or meditation on cortisol levels have shown promising results, but they often lacked sufficiently large samples or active control groups (e.g., Engert et al. 2018; Singer and Engert 2019). This study aims to investigate the effects of Yoga Nidra on both psychological outcomes (stress, sleep, and well‐being) and biological markers (diurnal cortisol patterns), extending previous findings with a structured randomised controlled trial (Moszeik et al. 2022).

This study builds on previous research by examining the effects of an 11‐min and a 30‐min version of Yoga Nidra on stress, sleep, well‐being, and diurnal cortisol levels. Additionally, it compares Yoga Nidra to an active control condition (music intervention) and a waitlist control group (WC) to isolate its specific effects. The primary objectives were to:

1. Assess whether Yoga Nidra reduces stress, anxiety, depression, and improves well‐being and sleep quality compared to a waitlist control.
2. Examine whether Yoga Nidra leads to measurable changes in diurnal cortisol patterns.
3. Compare the effects of a short (11‐min) versus a long (30‐min) Yoga Nidra practice.

By addressing both psychological and physiological outcomes, this study aims to contribute to the growing body of evidence on meditation‐based interventions and their potential for stress management and health promotion.

## 2 Yoga Nidra

The term Yoga Nidra can refer both to the method and the state of meditation. Recently, Yoga Nidra has gained recognition in the business world and among executives under the name ‘non‐sleep deep rest’ (NSDR), a term coined by Andrew Huberman (Jackson 2022). Sweet (2022, 13. april) describes NSDR, or Yoga Nidra, as a ‘next‐generation power nap’ that induces a sleep‐like state, allowing the brain to deeply relax in a short amount of time. This effect can be relaxing and energising, and may also positively influence the brain's neuroplasticity (e.g., Winter, as cited in Sweet 2022, 13. april). The brain's ability to develop and change can be influenced by positive stimuli, and is correlated with improved self‐regulation. This encompasses aspects such as attention control, emotion regulation, and self‐knowledge (Tang et al. 2015).

Another key mechanism in Yoga Nidra is mindfulness, a specific form of attention control. Kabat‐Zinn (2003) defines mindfulness as intentional, momentary, and nonjudgmental. During meditation, mindfulness helps centre the mind on the present moment and manage attention (Sedlmeier et al. 2012). Current research suggests that Yoga Nidra can enhance mental, physical, and emotional relaxation, potentially compensating for lack of sleep (e.g., Sharpe et al. 2021). A PET study found that Yoga Nidra meditators could regulate or stimulate consciousness at a synaptic level (Kjaer et al. 2002). During Yoga Nidra, practitioners often oscillate between waking and dreaming phases, resulting in an increased occurrence of alpha waves (8–14 Hz), which indicate a relaxed, awake state (Mandlik et al. 2002). In psychology, this state is known as the hypnagogic state and occurs during the transition to sleep (e.g., Sharpe et al. 2021). An increased occurrence of alpha waves can help release tension deeply and enhance recovery processes (e.g., Di Fronso and Bertollo 2021).

Emotion regulation can be achieved through confronting and potentially extinguishing emotions via meditation (e.g., Hölzel et al. 2011). This process allows feelings to be observed and examined, enabling the meditator to distance themselves from these emotions and re‐evaluate them. Yoga Nidra has been shown to increase cortical activity and improve control over the limbic system, leading to greater self‐awareness and reduced emotional excitability (e.g., M. Wheeler et al. 2017). This is also reflected in an increased ability to control one's actions, as evidenced by differentiated cortical activity in young adults with extensive yoga and meditation experience (Lou et al. 1999). The altered perception during Yoga Nidra leads to higher dopamine release, which can enhance drive and motivation (e.g., Lou et al. 2011). Additionally, meditation can increase self‐knowledge by helping practitioners adopt a meta‐perspective and alter automatic self‐assessments (Hölzel et al. 2011). This non‐evaluative observation allows for cognitive restructuring of one's perspective, recognising and changing reaction patterns. This process can also affect how one identifies with thoughts and feelings, enabling them to arise and be perceived without overwhelming the mind (e.g., Ardelt and Grunwald 2018). This process is also central to Acceptance and Commitment Therapy, where it is known as *cognitive defusion* (e.g. Hayes et al. 2016). Techniques in this approach aim to prevent negative thoughts or events from merging with the self, thus avoiding situations where thoughts like ‘I am fat, dumb, or ugly’ dominate one's self‐concept. Another element of Yoga Nidra is the *sankalpa*, a Sanskrit term meaning resolution or seed. This involves mentally repeating a simple, positive sentence at the beginning and end of the meditation (e.g.: ‘I am calm and relaxed’). This mental repetition is similar to autosuggestion or self‐affirmation, and it can be particularly effective when performed in a relaxed state (Satyananda 2009). Through the repetition of positive affirmations, the mind learns to form new habits and patterns, which can be seen as a reactive form of self‐regulation (Myga et al. 2022).

### 2.1 Extant Research on the Effectiveness of Yoga Nidra

‘Inner peace has become rare’ (Lattmann, 2011; as cited in Volk 2011). Of the health impairments resulting from stress, sleep disturbances have increased the most in recent years (BAuA 2020). In our previous study (Moszeik et al. 2022), we showed that just 11 min of Yoga Nidra improved subjectively estimated sleep quality and reduced daytime sleepiness, though the effects were small. In a literature review by Moszeik (2023), which included 45 studies on the effectiveness of Yoga Nidra, only 13 studies employed a randomised group comparison, and of those, only 5 included an active control group (cf. Supporting Information S1: Table S1). Another significant limitation in previous studies is the lack of information about the content and precise process of the Yoga Nidra meditation. For better comparability, a detailed description of the meditation process is essential. Compared to active controls, Yoga Nidra shows larger increases in positive affect and larger reductions in total cortisol concentration compared to progressive muscle relaxation and listening to audiobooks, although the respective studies did not report effect sizes (Borchardt et al. 2012). Compared to acupuncture, Yoga Nidra shows a more pronounced reduction in stress (M. S. Wheeler et al. 2018). When compared to physical yoga, practicing Yoga Nidra alone has an equally beneficial effect on heart rate, as shown in a small sample of 20 students (Markil et al. 2012). Compared to breathing exercises, participants practicing Yoga Nidra performed better in reducing high blood pressure, BMI (body mass index), and anxiety, as well as in relaxing heart rate and respiratory rate (Manik and Gartia 2016). Furthermore, a comparison with light physical work showed an increase in memory performance in the Yoga Nidra group, although the sample was very small (*n* = 12). In a sleep hygiene study with a small sample of 29 participants, Yoga Nidra positively influenced sleep duration (Gutman et al. 2016). Finally, Li et al. (2019) showed that Yoga Nidra had similarly positive effects on pain reduction and increased satisfaction compared to a music intervention. Wahbeh and Nelson (2019) also used music as an active control and found a larger reduction in sleep disturbances for the Yoga Nidra intervention, though again the sample was very small (*n* = 29).

### 2.2 Research Questions

Based on the gaps in our current knowledge, the main goals of this study were to replicate the effects of the 11‐min short form of Yoga Nidra found in a previous study (Moszeik et al. 2022) and to corroborate these effects using more sophisticated measures of stress and well‐being. This included considering an established biological stress parameter (salivary cortisol) and extending the meditation period from one to 2 months. Additionally, the follow‐up period was extended from 6 weeks to 3 months to assess the longer‐term effects of the intervention. Finally, the effects of Yoga Nidra were tested against an active control group (a music intervention). We specifically hypothesised that:

1. Compared to a waitlist control group:
  - (1a) Both the 11‐min and 30‐min forms of Yoga Nidra will lead to a significantly higher reduction in stress, cortisol levels, sleep disturbances, and negative affect, as well as a higher increase in mindfulness, life satisfaction, and positive affect.
  - (1b) The effects described in hypothesis (1a) will persist when compared to an active control group with a music intervention.
2. The 30‐min form of Yoga Nidra will lead to greater effects than the 11‐min form with respect to all criteria examined.

## 3 Method

### 3.1 Study Design

This work is an experimental longitudinal study conducted online. All surveys were created using Questback's Unipark programme. The study was carried out according to the recommendations and ethical guidelines of the German Society for Psychology and received a positive ethics vote from the University of the Bundeswehr, Munich. All participants took part anonymously and voluntarily, with the option to withdraw from the study at any time without any disadvantages. 3 online questionnaire surveys were conducted between June 2018 and January 2019 to examine the short‐ and longer‐term effects of Yoga Nidra (cp. Supporting Information S1: Table S2). The study design is based on a 4 × 3 factorial level combination for the factors of group and repeated measures. The primary objective was to assess psychological (stress, well‐being, sleep) and biological (cortisol) effects of Yoga Nidra, compared to an active control (music) and waitlist control group.

### 3.2 Sample

Participants in the present study registered via flyers, online advertising, and newsletters distributed at universities, medical practices, yoga studios, and on online platforms such as Facebook, LinkedIn, and Instagram. Inclusion criteria were: (a) minimum age of 18 (b) voluntary participation, (c) sufficient German language skills, and (d) internet access.

Exclusion criteria were: (a) current glucocorticoid medication (*n* = 6), (b) incomplete group assignment (*n* = 3), and (c) insufficient adherence to the saliva sampling protocol (e.g., incorrect timing). These criteria were evaluated at registration and/or during data validation prior to analysis. During the registration phase, a total of 426 subjects were randomly assigned to 1 of 4 groups: EG1, an experimental group with 11 min of Yoga Nidra (*n* = 112); EG2, an experimental group with 30 min of Yoga Nidra (*n* = 100); AC, an active control group with 10 min of music (*n* = 99); and WC, a waitlist control group without intervention (*n* = 115). Between registration and the first survey, there was a dropout of 61 subjects, resulting in a total of 365 participants who took part in the study. The a priori power analyses to compute the required sample size were conducted using G*power 3.1 software. These analyses indicated that a sufficient number of participants (344) were needed to achieve an actual power of 0.80 and to detect small effect sizes (0.20), based on the literature (one‐tailed alpha of 0.05).

Of the 362 participants, 101 were in the short meditation group (EG1; 27.9%), 80 were in the long meditation group (EG2; 22.1%), 74 were in the active control group with music (AC; 20.4%), and 107 were in the waitlist control group without any intervention (WC; 29.6%). At the end of the first survey, participants in the intervention groups were informed that their intervention period would be from July to August 2018. Participants in the WC were informed that several intervention phases would take place between July 2018 and January 2019, and they would be notified when their meditation period would begin. To ensure anonymity, participants created a personal, 6‐digit pseudonymization code, which was used to merge the survey data.

### 3.3 Measures

All measures are listed in Supporting Information S1: Table S3 and were collected through online questionnaires. An exception is the analysis of salivary cortisol, which is described in more detail afterwards. Salivary cortisol was also collected at all three measurement points, but participation was optional. At the first measurement point, demographic data were also collected to later control for the effects of meditation (Supporting Information S1: Table S4).

### 3.4 Stress

Stress was measured using the 12‐item screening scale of the Trier Inventory for Chronic Stress (SSCS, TICS; Schulz et al. 2004), which captures a global measure of stress (sample item: ‘Experience that everything I have to do is too much’). Participants reported mean stress levels at t1 that were significantly higher than those of a representative German sample (Petrowski et al. 2012) with *t* (2674) = −17.05, *p* < 0.0001.

### 3.5 Well‐Being

According to Diener et al. (1999), well‐being is composed of cognitive and affective components: high life satisfaction, high positive affect, and low negative affect. The present study considers life satisfaction, rumination (worrying about past events), anxiety, and depression. These are divided into different aspects of affective feeling—anxiety addresses the physical state of arousal (excitement) and the experience of worrying about the future, while depression is associated with a lack of positive affect (euthymia) and a strong negative affect (dysthymia) (Renner et al. 2018). The choice of instruments in this study is based on a previous study (Moszeik et al. 2022) and aims to provide a more detailed understanding of the effects of Yoga Nidra according to these scales. Current research suggests that through Yoga Nidra and other meditation‐based interventions, affective areas of well‐being can be changed, particularly by reducing negative states of arousal.

### 3.6 Anxiety and Depression

First, the trait items of the German version of the State‐Trait Anxiety‐Depression Inventory (STADI; Laux et al. 2013) were presented. These 20 items not only assess anxiety and depression but also differentiate between emotionality (example item: ‘I feel fine’, reversed item) and worry (example item: ‘I worry about problems which could come up’), as well as euthymia (example item: ‘I am fun‐loving’) and dysthymia (example item: ‘I am dejected’) for recording depression. Participants rated how often they had a certain feeling or thought in the past 2 months using a 4‐point scale (1 = *almost never* to 4 = *almost always*). Completing the scale takes about 5–10 min. Higher values indicate higher anxiety or a greater tendency towards depression. Scores for euthymia were recoded to compute the total depression score. The participants' baseline scores at t1 were significantly higher than those of a representative German sample (Laux et al. 2013), with anxiety scores at *t* (3485) = −10.61, *p* < 0.0001, and depression scores at *t* (3485) = −5.01, *p* < 0.0001.

### 3.7 Rumination

Since a differentiated consideration of affective and cognitive well‐being is central to the present study, the German version of the Rumination scale from the Rumination‐Reflection Questionnaire (RRQ; König 2012; original by Trapnell and Campbell 1999) was also used to examine the level of worrying more closely. The scale consists of 12 items related to typical thoughts participants have (example item: ‘I tend to worry or think about things that happened to me for a very long time’; 1 = *completely disagree* to 5 = *completely agree*). Participants in the present study reported mean rumination scores at t1; a comparison with a representative German sample is not available.

### 3.8 Satisfaction With Life

Finally, as a further cognitive variable of well‐being, life satisfaction was measured using the German version of the Satisfaction with Life Scale (SWLS; Diener et al. 1985; German version by Schumacher 2003). This scale consists of five items and takes about 1–2 min to complete (1 = *completely disagree* to 7 = *completely agree*; example item: ‘I am satisfied with my life’). Participants' scores did not differ significantly from those of a representative German sample (Glaesmer et al. 2011), with *t* (2854) = 0.77, *p* = 0.44.

### 3.9 Sleep Quality

The German version of the Pittsburgh Sleep Quality Index (PSQI; Buysse et al. 1989; German version by Riemann and Backhaus 1996) was used to measure sleep quality across six components: subjectively assessed sleep quality (1 item), sleep latency (2 items), sleep duration (1 item), sleep disturbances (9 items), consumption of sleeping pills (1 item), and daytime sleepiness (2 items). The total of 16 items could be completed in about 5–10 min (example item for daytime sleepiness: ‘Over the past 4 weeks, have you had problems completing usual everyday tasks with enough energy?’; 0 = *no problems* to 3 = *major problems*). A comparison with a representative sample is not possible, as Buysse et al. do not provide mean values for comparison.

### 3.10 Mindfulness

The level of mindfulness was measured using the short German version of the Five‐Facet Mindfulness Questionnaire (FFMQ Baer et al. 2006; FFMQ‐15 Baer et al. 2012; German version FFMQ‐D by Michalak et al. 2016). This questionnaire assesses mindfulness in a detailed manner and is divided into five facets, each with three items in the short form used (1 = *almost never* to 5 = *almost always*). The facets are: Observing (example item: ‘When I shower or bathe, I am aware of the feeling of water on my body.’), describing (example item: ‘I can put my feelings into words well.’), acting with awareness (example item: ‘I am easily distracted.’; recoded), acceptance without judging (example item: ‘I tell myself that I shouldn't feel what I'm feeling.’; recoded) and non‐reactivity (example item: ‘If I have distressing thoughts or ideas, I can distance myself from them take and am aware of the thoughts or ideas without being overwhelmed by them.’). Participants in the present study reported average mindfulness values at t1. A comparison with a representative German sample is not possible due to the lack of comparative data at the time of the study. Since the facet of observing can be interpreted differently by individuals with varying levels of meditation experience, the five facets are considered individually rather than combined into an overall mindfulness score for this pre‐post intervention (Gu et al. 2016). Individuals with less experience might report observing more, but this may reflect neutral or even negative perceptions rather than a nuanced understanding of mindfulness.

### 3.11 Salivary Cortisol Analyses

Cortisol serves as a biomarker for the activity of one of the most critical stress systems, the HPA axis (e.g., G. E. Miller et al. 2007). Deviations from the normal daily rhythm of cortisol can indicate the impact of long‐term exposure to stress. The present study complements findings from online questionnaires with biological data in the form of salivary cortisol. Participants collected five saliva samples at home across two consecutive days, following a clearly structured collection schedule with labelled Salivette tubes and time sheets. Samples were immediately stored in the participants' home freezers (−18°C) until they were returned via prepaid postal shipment using insulated envelopes. Samples were then shipped in batches to the biopsychological laboratory at the Chair of Health Psychology at Friedrich‐Alexander University of Erlangen‐Nuremberg. Upon arrival, the samples were immediately stored at −30°C until batch processing. All samples were analysed blinded and within a standardised time frame to avoid storage‐related bias. This collection and storage approach is in line with standard recommendations for ambulatory salivary cortisol research (e.g., Rohleder et al. 2009). Diurnal profiles were collected on two consecutive days, with five saliva samples taken each day. This approach provides a robust recording of cortisol release variations due to daily stressors or activities (e.g., G. E. Miller et al. 2007). The sampling schedule was as follows:first sample (S1): immediately upon awakening, second sample (S2): 30 min after awakening, third sample (S3): 4 h after awakening, fourth sample (S4): 9 h after awakening, fifth sample (S5): 13 h after awakening or before bedtime, whichever comes first. In the present study, samples from 229 participants were analysed in the laboratory at University of Erlangen‐Nuremberg. Cortisol concentrations were determined by chemiluminescence immunoassay (CLIA, IBL, Hamburg, Germany) in duplicates. Two established parameters were used to assess changes in cortisol levels. To estimate total cortisol output over the measurement period, we computed the area under the curve with respect to ground (AUCg; Pruessner et al. 2003). AUCg reflects the total amount of cortisol and is used in this study as a comparative value between groups, representing the absolute cortisol concentration in each group over time. To estimate diurnal cortisol variation, we computed linear cortisol slopes, which are crucial for interpreting cortisol concentrations. We focused on two primary parameters:

1. Cortisol Awakening Response (CAR): This measures the increase in cortisol levels between the first and second diurnal measurement points, typically reflecting an upward slope.
2. Diurnal Flattening: This represents the decrease in cortisol levels from the first to the fifth diurnal measurement point, often seen as a downward slope. Values of cortisol between 5 and 23 nmol/L are common from 8 a.m. to 12 p.m., and these values should decrease throughout the day, reaching their lowest in the late evening. Less steep diurnal flattening is generally associated with lower well‐being (G. E. Miller et al. 2007).

For accuracy, if the specific time for sample collection was not provided on the timesheet, a default value of 30 min was applied (*n* = 49). Samples taken earlier than 15 min or later than 60 min from the scheduled time were excluded from the analysis (*n* = 40), as measurements must adhere to specified time frames to align with diurnal cortisol release patterns. Rohleder et al. (2009) suggest that taking at least three, ideally five, measurements per day over 2‐3 consecutive days is best practice. Additionally, samples from individuals taking medications such as glucocorticoids were excluded due to their direct impact on cortisol levels, which could confound the data (*n* = 6). Since no representative German sample is currently available for comparison, the cortisol values cannot be related to a standardised reference. There are also no established standard values for interpreting AUCg and slopes. Instead, the data are considered in terms of relative increases or decreases and are analysed specifically within the context of the collected sample.

In the present study, the mean values of the cortisol parameters at t1 were as follows: Area Under the Curve with respect to ground (AUCg): 438.50 nmol/min/L (SD = 101.98, *n* = 141); Cortisol Awakening Response (CAR): 0.261 nmol/h/L (SD = 0.363, *n* = 135); Diurnal Flattening Slopes: −0.044 nmol/h/L (SD = 0.014, *n* = 141). To account for outliers and ensure data normality, all cortisol values were log‐transformed as recommended by Rohleder et al. (2009).

### 3.12 Procedure and Experimental Conditions

The three different interventions were delivered to participants via audio files sent by email. Each participant was responsible for independently implementing their assigned intervention over the course of the 2‐month intervention period. In addition to the audio files, participants received a document to log their meditation sessions, and bi‐weekly informational emails were sent to provide guidance on the meditation practice, serve as reminders, and offer motivational support (e.g., referring the personal intention, speed of the recording and visualisations). To further encourage participation, a short video clip was provided, aimed at increasing engagement and motivation (cf. Manthey et al. 2016). While no direct supervision was provided, participants were encouraged to reach out via email with any questions or concerns. Variables were assessed before the intervention, immediately after the 2‐month meditation period, and again 3 months later. The Yoga Nidra intervention was based on Satyananda Yoga Nidra protocols and adapted into 2 standardised audio versions (11‐min and 30‐min) with guidance from experienced Yoga Nidra practitioners.

#### 3.12.1 EG1—11 Minutes Yoga Nidra (Short Version)

The first experimental group (EG1) practiced the 11‐min short form of Yoga Nidra, which had been developed and evaluated in a prior study (Moszeik et al. 2022). This abbreviated version consisted of:

- A brief body scan
- Observation of the breath for approximately 20 s
- Setting a personal intention (*sankalpa*)
- Observation of thoughts and feelings

#### 3.12.2 EG2—30 Minutes Yoga Nidra (Long Version)

In the 30‐min Yoga Nidra group (EG2), participants were provided with an extended version of the Yoga Nidra practice, which included the following components:

- The body scan was extended
- The breath awareness component was also prolonged
- Setting a personal intention (*sankalpa*)
- Observation of thoughts and feelings
- Chakra Perception: Participants engaged in an exercise focussing on the perception of energy centres or nerve pathways along the spine, known as chakras in yoga (Sanskrit for ‘wheel’). Each of the seven chakras was introduced with a specific quality (e.g., strength), which participants were guided to visualise mentally. This exercise lasted approximately 5 min for all chakras. Participants reflected on the theme associated with each chakra, such as the meaning of strength and its presence in the current moment and observed their reactions to this reflection.
- Balancing Sensations: Additional exercises were included to balance various sensations, such as feeling light or heavy and observing positive and negative thoughts without judgement.

Both Yoga Nidra audio files were based on the teachings of Satyananda Yoga Nidra and the Bihar School of Yoga and included a personal intention or *sankalpa*, which was repeated at the beginning and end of the meditation. The audio files and instructional video were provided in German without background music and are available upon request.

#### 3.12.3 AC—10‐Min Music Intervention

For the active control group (AC) in this study, a 10‐min music intervention was employed, derived from the SOM programme (Santulan OM Meditation) as described by També (2016). This intervention is designed to enhance satisfaction and reduce tension and anxiety. According to També (2016), the application of this music does not require prior experience and can aid in relieving tension. The theoretical basis of this intervention posits that the sound vibrations influence the body's fluids, potentially easing tension and facilitating a meditative state. Additionally, it is suggested that this intervention positively impacts sleep. The efficacy of music interventions for reducing stress, improving sleep, and enhancing well‐being has been supported by various studies (e.g., Harmat et al. 2008).

### 3.13 Statistical Analysis for Hypothesis Testing

Statistical analyses were conducted using IBM SPSS Statistics 24.0 and Onyx (von Oertzen et al. 2015). To test the hypotheses, we employed correlation and variance analyses within structural equation models, which allowed for the inclusion of missing values at individual survey time points. Given the directed nature of the hypotheses, one‐sided *p*‐values and one‐sided likelihood‐ratio values (LR) were reported. Effect sizes were quantified using Cohen's d. Due to the sufficiently large sample size, error variances were calculated under the assumptions of normal distribution and homogeneity. Pretest differences were assessed using a likelihood ratio test in Onyx. Mean values between groups were compared, and significant differences were considered for subsequent analyses. This approach minimised error variance and ensured that true differences between groups were accurately identified.

### 3.14 Analyses of Variance With Repeated Measures

Data from all three measurement points were analysed using a variance‐analytical repeated measures design. This analysis was conducted within a structural equation model (SEM) to accommodate missing data effectively through Full Information Maximum Likelihood (FIML) estimation. The SEM, illustrated with stress data in Supporting Information S1: Figure S2, compares two experimental groups: EG1 (group code ‘4’) and EG2 (group code ‘5’).

A multi‐level structure was established for the repeated measures, with comparisons made alternately between two of the four groups. This approach allows the ANOVA model to be implemented within the SEM framework. Given that the missing data were assumed to be missing at random, FIML estimates were expected to be minimally biased. For the interaction effect between the follow‐up period and the respective intervention group, the a posteriori probability was also assessed, indicating whether the observed interaction effect aligns with the expected intervention effect in the intervention group. Furthermore, baseline comparability between groups was accounted for within the SEM framework, ensuring that potential differences were statistically controlled for in the model estimation. Therefore, separate ANOVAs or chi‐square tests were not conducted, as the SEM approach inherently addresses baseline variations during parameter estimation.

### 3.15 Pre‐Registration

The study was not formally pre‐registered in a public repository. However, all core elements of the study—including group assignment, intervention conditions, hypotheses, outcome variables, and measurement timepoints—were specified in advance and remained unchanged throughout data collection and analysis. Additional exploratory moderation analyses were conducted post hoc and are clearly labelled as such in the Results and Discussion sections.

## 4 Results

Descriptive statistics, including means and standard deviations for all measures across the three measurement points in each of the four groups, are presented in Supporting Information S1: Table S5. The following analyses refer to a total sample of 362 participants, including 272 women (80.7%), 64 men (19%), and 1 diverse (25 did not provide a statement). The mean age was 40.41 years (median: 39, range: 19–80), with most participants (65.9%) being married or in a permanent partnership. The overall sample had a very high level of education (e.g., participants with a university degree = 45.6%). To illustrate the flow of participants, we included a CONSORT Flow Chart as supplemental online material (Supporting Information S1: Figure S1). Regarding dropout rates between groups and over time, most participants dropped out of the WC group at t1 (*n* = 18, 16.8%). At t2, the highest dropout was observed in EG1 (*n* = 31, 31.6%), followed by AC (*n* = 19, 26.7%), and then EG2 (*n* = 16, 20.3%). A chi‐square test comparing dropout rates between groups and over time (not dropped out vs. dropout between t1 and t2 vs. dropout between t2 and t3) did not reveal any significant differences (*p* = 0.078 between t1 and t2, and *p* = 0.138 between t2 and t3). While we did not systematically record specific dropout reasons, we have ensured that the CONSORT flowchart (Supporting Information S1: Figure S1) accurately represents participant retention across all study phases, including attrition at each time point (t1, t2, t3).

### 4.1 11‐min Yoga Nidra Short Form versus Waitlist Control Group

As detailed in Supporting Information S1: Table S6, the interaction effect confirmed the hypothesis, showing at least a 96% a posteriori probability under a flat prior (Bayes factor > 28) for measures of stress, anxiety, depression, rumination, and life satisfaction. For mindfulness, significant effects were observed only for the facets of non‐reactivity and acceptance without judgement. Compared to the WC group, the 11‐min Yoga Nidra short form resulted in a significant reduction in stress, anxiety, depression, and rumination. Additionally, there was an almost significant reduction in sleep disturbances and a notable increase in life satisfaction, along with improvements in the two aforementioned facets of mindfulness. The effect sizes for the 11‐min Yoga Nidra short form compared to the waitlist control group indicate improvements ranging from 8% to 16% of the standard deviation, corresponding to effect sizes of *d* = 0.08–0.16. The 95% confidence intervals (CIs; equivalent to 95% Bayesian intervals) for the significant effect sizes were entirely above or below zero, with the exceptions of life satisfaction [‐0.01; 0.34] and sleep quality [‐0.18; 0.02]. For the other variables, no significant differences were found between the 11‐min Yoga Nidra short form and the waitlist control group. Thus, Hypothesis 1a was partially confirmed. For all significant results on dependent variables, except for sleep quality, the probability that the follow‐up effect will negate the interaction effect is below 9.26%. For sleep quality, this probability is 27.23%. Consequently, it can be concluded that the observed effects were largely maintained at follow‐up.

### 4.2 11‐Min Yoga Nidra Short Form versus Active Control Group

The comparison between the 11‐min Yoga Nidra short form and the active control group (AC) using music intervention revealed significant findings primarily for depression. As shown in Supporting Information S1: Table S7, the interaction effect supports the hypothesis with at least 96% a posteriori probability under a flat prior, specifically for depression (Bayes factor = 148.26). The short form of Yoga Nidra resulted in a significant reduction in depression compared to the AC, with an improvement of approximately 13% relative to the standard deviation (SD), corresponding to an effect size of *d* = 0.13. The 95% confidence interval (CI) for the depression effect size was entirely on one side of zero, indicating a robust finding. Notably, depression levels in the AC increased from t1 to t2.

For other variables, no significant differences were observed between the short Yoga Nidra form and the music intervention. Thus, Hypothesis 1b was confirmed only for depression. The probability that the follow‐up effect overrides the interaction effect is 2.62%, indicating that the effects of the Yoga Nidra short form on reducing depression were maintained at follow‐up.

### 4.3 Active Control Group versus Waitlist Control Group

A significant interaction effect was found for life satisfaction (*est.* = 0.24, *se* = 0.12, *LR* (1) = 4.17, *p* = 0.020, Bayes factor = 50.41). The music intervention led to a significant increase in life satisfaction compared to the WC, with an improvement of about 7% relative to the SD, corresponding to an effect size of *d* = 0.07. The 95% CI was completely above zero. For other variables, no significant differences were found between the AC and WC. The probability that the follow‐up effect negates the interaction effect is 99.64%, suggesting that the effect of the music intervention on increasing life satisfaction is not sustained over time.

### 4.4 11‐Min Yoga Nidra Short Form versus 30‐Min Yoga Nidra Long Form

As shown in Supporting Information S1: Table S8, the interaction effect, conforming to the hypothesis, was found with at least 96% a posteriori probability under a flat prior only for the facet ‘acting with awareness’ (Bayes factor = 32.14). The 30‐min Yoga Nidra resulted in a significant increase in ‘acting with awareness’ compared to the short form, with an improvement of about 10% relative to the standard deviation (SD), corresponding to an effect size of *d* = 0.10. The 95% CI for this effect size is almost entirely on one side of zero [−0.01; 0.44]. For other variables, no significant differences were found between EG2 (30‐min form) and EG1 (11‐min form), even though the change in total cortisol approached significance. Descriptive data generally indicate a stronger influence of EG2, but these tendencies were not statistically confirmed. The probability that the follow‐up effect overruled the interaction effect for ‘acting with awareness’ was 55.13%. Thus, it can be concluded that the effect of the 30‐min Yoga Nidra compared to the short form is only partially retained at the follow‐up examination. To further validate the effectiveness of EG2 (30‐min Yoga Nidra), comparisons with the active control group (AC) and the waitlist control group (WC) were also conducted for all dependent variables:

### 4.5 30‐Min Yoga Nidra Long Form versus Active Control Group

Significant improvements were observed for stress (*est*. = −0.20, *se* = 0.07, *LR* (1) = 2.97, *p* = 0.042), depression (*est.* = −0.15, *se* = 0.09, *LR* (1) = 3.06, *p* = 0.040), rumination (*est*. = −0.17, *se* = 0.10, *LR* (1) = 2.70, *p* = 0.050), and the cortisol awakening response (*est*. = −0.20, *se* = 0.12, *LR* (1) = 2.80, *p* = 0.047). These effects are significant in a one‐sided likelihood‐ratio test against the null hypothesis (Bayes factor ≥ 16.82). Compared to the AC, 30 min of Yoga Nidra led to a significant reduction in stress, depression, rumination (each approx. 9% improvement with respect to the SD) and a flatter rise in the cortisol wake‐up reaction (6% improvement in terms of the SD). The corresponding effect sizes ranged between *d* = 0.06–0.09. The 95% CIs of the effect sizes are not completely above or below zero for any of the variables but are closest for depression [−0.32; 0.02]. For stress, depression, and rumination, the probability that the follow‐up effect negates the interaction effect is less than 5.29%. For the cortisol awakening response, this probability is 41.55%. From these findings, it can be concluded that the effects of the 30‐min Yoga Nidra intervention on stress, depression, and rumination are largely retained at follow‐up, while the effect on the cortisol awakening response is less stable.

### 4.6 30‐Min Yoga Nidra Long Form versus Waitlist Control Group

There were significant reductions in stress (*est*. = −0.35, *se* = 0.10, *LR* (1) = 12.33, *p* < 0.001), anxiety (*est*. = −0.15, *se* = 0.08, *LR* (1) = 3.77, *p* = 0.026), depression (*est*. = −0.12, *se* = 0.07, *LR* (1) = 2.79, *p* = 0.047), rumination (*est*. = −0.31, *se* = 0.10, *LR* (1) = 9.25, *p* = 0.001), and sleep disturbances sleep (*est*. = −0.13, *se* = 0.06, *LR* (1) = 5.15, *p* = 0.012). Additionally, there was a significant increase in the observing facet of mindfulness (*est.* = 0.26, *se* = 0.11, *LR* (1) = 5.54, *p* = 0.009) and a significant reduction in the slope of the cortisol awakening response (*est*. = −0.25, *se* = 0.12, *LR* (1) = 4.30, *p* = 0.019). These effects were significant in a one‐sided likelihood‐ratio test against the null hypothesis (Bayes factor ≥ 8.85).

Compared to WC, 30 min of Yoga Nidra led to a significant increase in observing (12%) and significant reductions in stress (19%), anxiety (10%), depression (9%), rumination (16%), sleep disturbances (12%), and a flatter slope of the cortisol awakening reaction (8%), corresponding to effect sizes of *d* = 0.08–0.19. The 95% CIs of the effect sizes are completely above or below zero for stress, rumination, sleep disturbances, observing, and the cortisol awakening response: The CIs are almost entirely below zero for anxiety [−0.31; < 0.01] and depression [−0.27; 0.02]. For stress, anxiety, depression, rumination, sleep disturbances, and the cortisol awakening response, the probability that the follow‐up effect negates the interaction effect is less than 11.87%. For observing, this probability is 17.44%. Therefore, it can be concluded that the effects of the 30‐min Yoga Nidra intervention are largely retained at the follow‐up examination for the variables mentioned when compared to the waitlist control group.

### 4.7 Additional Exploratory Results

At t1, the participants in the study reported moderate levels of experience with mindfulness and/or meditation (*M* = 3.11, SD = 1.10; *N* = 337, with 1 = *none* and 5 = *very much*). Exploratory analyses revealed that the effects of the 11‐min Yoga Nidra (EG1) were significantly moderated by prior meditation experience concerning depression (*est*. = −0.09, *se* = 0.05, *LR* (1) = 3.91, *p* = 0.048) and the describing facet of mindfulness (*est*. = 0.19, *se* = 0.07, *LR* (1) = 6.54, *p* = 0.010). Greater prior experience was associated with a larger decrease in depression and a larger increase in the describing facet.

In the 30‐min Yoga Nidra group (EG2), participants with less prior experience showed more benefits; less prior experience correlated with a greater increase in life satisfaction (*est*. = 0.28, *se* = 0.12, *LR* (1) = 5.36, *p* = 0.020).

For the 11‐min Yoga Nidra group (EG1), more regular practice was linked to significant decreases in total cortisol concentration (*est*. = −23.45, *se* = 13.60, *LR* (1) = 2.73, *p* = 0.049), and steeper slopes in the cortisol awakening response (*est.* = 0.16, *se* = 0.08, *LR* (1) = 3.36, *p* = 0.033) and cortisol flattening (*est*. < −0.01, *se* < 0.01, *LR* (1) = 42.10, *p* < 0.001). These findings indicate that the frequency of Yoga Nidra practice significantly impacted biological stress markers (cf. Supporting Information S1: Figures S1‐S3).

In the 30‐min Yoga Nidra group (EG2), increased frequency of practice was associated with greater decreases in rumination (*est*. = −0.13, *se* = 0.08, *LR* (1) = 2.92, *p* = 0.044), increases in non‐reactivity (*est*. = 0.18, *se* = 0.11, *LR* (1) = 2.80, *p* = 0.047), and increases in the ‘acting with awareness’ facet (*est*. = 0.16, *se* = 0.08, *LR* (1) = 4.00, *p* = 0.023). However, the total cortisol concentration fell less steeply (*est*. = 44.82, *se* = 17.32, *LR* (1) = 6.02, *p* = 0.007), and the slope of the cortisol awakening response flattened out less steeply (*est*. = 0.19, *se* = 0.09, *LR* (1) = 4.18, *p* = 0.020).

In the active control group (AC), the frequency of the 10‐min music intervention was positively related to an increase in non‐reactivity (*est*. = 0.22, *se* = 0.11, *LR* (1) = 4.28, *p* = 0.019). Prior experience also correlated positively with the frequency of meditation practice (*r* = 0.242). A descriptive overview of the frequency of practice in all three intervention groups is provided as supplementary online material (Supporting Information S1: Table S9). Additionally, participants were asked if they continued to use the intervention after t2. Of the 147 participants who answered this question at t3, 63 (42.9%) reported no longer using the intervention, while 84 (57.1%) continued to use it. Notably, most continuations of the intervention beyond the official period occurred in EG1. Further exploratory findings can be found at online supplemental material (Supporting Information S1: Table S10).

## 5 Discussion

The present study aimed to examine the effects of Yoga Nidra relative to an active control group (music intervention), incorporating an established biological stress parameter (salivary cortisol). Additionally, it sought to replicate and extend the findings of the 11‐min Yoga Nidra short form compared to a waitlist control group (WC) by including a longer meditation and intervention period of 2 months instead of one.

### 5.1 Effects of the 11‐Min Yoga Nidra

The effects of the 11‐min Yoga Nidra short form were successfully replicated. The most significant impact was observed in the reduction of anxiety (*d* = −0.16), indicating a stable effect of the short form on affective well‐being. Notably, all three cortisol measures showed significant changes when the frequency of meditation was considered: total cortisol levels decreased, and the slopes of the cortisol awakening response and evening flattening became steeper. These changes suggest greater activation in the morning and greater relaxation in the evening, with steeper flattening slopes being associated with greater well‐being (G. E. Miller et al. 2007). However, these findings are based on correlational analyses and should not be interpreted causally. In terms of mindfulness, the short form of Yoga Nidra primarily influenced facets associated with distancing from stressful thoughts (non‐reactivity) and accepting one's thoughts and feelings (acceptance without judgement). Other aspects of mindfulness, such as articulating feelings (describing), awareness of sensations (observing), and focused action (acting with awareness), were not significantly affected.

### 5.2 Comparison With Active Control (AC)

Compared to the AC, 11 min of Yoga Nidra significantly impacted depression (*d* = 0.13), with the effect being almost entirely retained at follow‐up (97.38%). This effect might also be attributed to an increase in depression within the AC group from pre‐to post‐test. Specifically, the component of euthymia (reverse scored as anhedonia) showed a significant increase compared to the AC, highlighting Yoga Nidra's potential to enhance positive affect (cf. Rani et al. 2011). While the music intervention served as an active control, the more structured nature of Yoga Nidra may have facilitated greater emotional regulation, contributing to the observed reduction in depressive symptoms. The increase in depression scores in the AC group may be attributed to expectation effects, frustration, or disappointment due to not receiving a structured meditation practice like Yoga Nidra. This aligns with findings from previous mindfulness control studies, where passive interventions (e.g., music) sometimes lead to nocebo‐like effects (Zeidan et al. 2010).

The AC, when compared to the WC, demonstrated a small but significant effect on life satisfaction (*d* = 0.07). This suggests that to improve life satisfaction, a different approach may be needed, one that requires less cognitive effort than Yoga Nidra. Music, as a less cognitively demanding intervention, might be more effective in this regard. However, the effect of the music intervention did not persist at follow‐up (0.36%). The lack of significant differences between AC and WC suggests that the music intervention did not provide substantial additional benefits over no intervention. This could be due to:

1. The passive nature of the music condition, which lacks structured guidance or cognitive engagement.
2. Potential self‐selection biases, where participants who expected greater benefits from an active intervention may have responded differently.
3. Ceiling effects in stress reduction, as participants in both groups may have engaged in other personal relaxation strategies.

### 5.3 Comparison Between Short and Long Forms of Yoga Nidra

The comparison between the 11‐min and 30‐min forms of Yoga Nidra revealed that the longer session significantly improved ‘acting with awareness’ (*d* = 0.10), with the effect partially retained at follow‐up (44.87%). This suggests that longer sessions can enhance attentive actions characterised by focus and low distractibility (cf. Michalak et al. 2016). ‘Acting with awareness’ is considered a critical facet of mindfulness, particularly effective for mental health (Heeren et al. 2021). Contrary to previous assumptions, the longer meditation did not have a greater impact on cognitive components of well‐being, such as life satisfaction and rumination. This suggests that less cognitive interventions, like music, might better stimulate cognitive well‐being, warranting further investigation in future studies.

### 5.4 Effects of the 30‐Min Yoga Nidra

When compared to the AC group, 30 min of Yoga Nidra significantly reduced depression and marginally affected stress, rumination, and the cortisol awakening response (*d* = 0.06–0.09). The confidence intervals for depression were notably more significant. Effects were largely maintained at follow‐up (58.45%–94.71%). Specifically, the impacts on stress, depression, and rumination were preserved, though cortisol changes were less sustainable. These results support Yoga Nidra's effectiveness in reducing negative affect, consistent with previous research (e.g., Wahbeh and Nelson 2019), and suggest a more relaxed morning start. This finding aligns with Pruessner et al. (1999), who noted that a less steep cortisol awakening response is associated with lower stress. Our study confirmed this both through subjective self‐reports and biological measures. However, when the long meditation was practiced more frequently, the slope of the cortisol awakening response became steeper, and total cortisol levels increased. This suggests that increased meditation may lead to greater activation, consistent with literature indicating that meditation can initially be relaxing but may become energising with regular practice (e.g., Prakashananda 2010).

Compared to the WC, 30 min of Yoga Nidra led to the greatest reduction in stress (*d* = −0.19). Frequent meditation also decreased rumination, increased non‐reactivity and ‘acting with awareness,’ and resulted in a smaller reduction in total cortisol and a higher cortisol awakening response.

Both long and short form of Yoga Nidra effectively reduced stress, anxiety, and depression, albeit with small effect sizes. Our results suggest that either frequent short sessions or longer sessions of meditation can induce biological changes. The direction of these changes—whether activation increases or decreases—should be assessed on an individual basis to determine what is most beneficial.

### 5.5 Strengths and Weaknesses

The strengths of this study include: (1) the experimental longitudinal design, which involved an active control group and a sufficiently large, diverse sample; (2) the use of cortisol as a biological stress marker in addition to subjective self‐assessments; and (3) the comprehensive assessment of mindfulness across five different facets. Notably, the salivary cortisol measures were conducted with one of the largest samples to date (*n* = 229) examining diurnal cortisol changes through a meditation‐based intervention with an active control group design across three measurement points. The effects observed on the cortisol awakening response are the first to be demonstrated in relation to mindfulness meditation and warrant replication in future research, particularly to verify changes in cortisol levels with different meditation lengths and frequencies. A key strength of the study is its online administration, which offers economic advantages. However, this approach also limits personal guidance and support during meditation training, which could be crucial, for instance, when implementing personal resolutions (e.g., Satyananda 1980). Myga et al. (2022) recommend using autosuggestion in experimental studies to improve comparability in future research.

While the observed effect sizes are small, they are consistent with previous mindfulness‐based interventions (Hülsheger et al. 2015; Sedlmeier et al. 2012). Even small effects can be meaningful in large‐scale interventions, especially when considering the low cost, accessibility, and minimal effort required for Yoga Nidra practice. It is essential to contextualise these small effects within a larger sample and meaningful framework: small effects are not inherently inferior to larger ones. As noted by Funder and Ozer (2019), even small effects can be important if they lead to substantial improvements in psychological and physiological well‐being. The cumulative effect, where repeated small impacts accumulate over time, underscores the significance of small effects in psychological research. Limitations of the study include unequal group sizes due to dropout between registration and the first measurement point, as well as dropout among the four groups throughout the study. Additionally, while cortisol analyses adhered to high standards, there is considerable variability in the field regarding collection and calculation methods. Laufer et al. (2022) have proposed a method to standardise cortisol collection and reporting, which could enhance comparability in future research.

Since our analysis was based on pre‐specified hypotheses and employed a structural equation modelling (SEM) approach with likelihood‐ratio tests, we did not consider an additional correction for multiple comparisons necessary (Perneger 1998; Schneider 2008). Instead, we focused on theoretically justified comparisons and prioritised non‐exploratory analyses.

## 6 Conclusion

Whether referred to as Yoga Nidra, non‐sleep deep rest, or another form of meditation, the nervous system benefits from every moment of relaxation. However, the current empirical evidence is insufficient to fully understand how different meditation forms work and for whom they are most effective. Promising research indicates that the impact of various positive interventions can be moderated by personality traits (cf. Talic et al. 2023). Future research should not only highlight the positive effects of meditation but also empirically determine under which individual conditions these effects are optimised. It is also worth exploring whether simply relaxing with music could be a viable alternative. The positive‐activity model (Lyubomirsky and Layous 2013) provides a useful framework for this investigation. It considers both individual characteristics (e.g., motivation and effort) and features of the activity itself (e.g., dosage and variety) to enhance well‐being through positive interventions. Further research using this model could help clarify how different factors contribute to the effectiveness of meditation and other positive practices.

## Conflicts of Interest

The authors declare no potential conflicts of interest with respect to the research, authorship, and publication of this article.

## Supporting information

Supporting Information S1

## Data Availability

The dataset generated and analysed during the current study is available from the corresponding author on reasonable request.
