# Supplementary material for: The Effects of an Online Yoga Nidra Meditation on Subjective Well‐Being and Diurnal Salivary Cortisol: A Randomised Controlled Trial
Source: Stress Health. 2025 May 15;41(3):e70049. doi: 10.1002/smi.70049 (PMC12080877; doi:10.1002/smi.70049)
Supplement: Supplementary file 1 — Supporting Information S1 [file SMI-41-e70049-s001.pdf]

Supplemental online material

Table S1

Current Empirical Evidence on the Effects of Yoga Nidra in Randomized or Non-Randomized but Controlled (Quasi-Experimental) Designs

Study	Design	Sample	Effects of Yoga Nidra	**Effect sizes**
Deuskar (2008)	RCT: 30x YN in 6 months	101 students	Decrease in test anxiety (emotionality) but not in worrying	n/a
Amita et al. (2009)	RCT: 3 months daily YN	41 diabetes patients (20 EG, 21 CG: only medication)	Changes in: blood levels, sweating, sleep, stress, palpitations, headaches	n/a
Deuskar (2011)	RCT: 30x YN over 1.5 months	95 low-income working mothers (42 EG, 53 CG)	Decline of physiological + psychological stress levels (SSSI, blood pressure and blood sugar, hemoglobin)	small to big
Rani et al. (2011, 2013)	RCT: 5x/week YN in 6 months	150 women with menstrual disorders (75 EG: medication plus YN and 75 CG: only medication)	Decline of pain, anxiety, depression; increase for well-being, general health (PGWI, 2011); Hormonal Balance (Hormone Profile, 2013)	n/a
Nassif et al. (2014)	RCT: 8 weeks YN (iRest)	9 Veterans with chronic pain (4 in EG, 5 in CG)	Decrease in pain intensity (VAS, DVPRS, PGIC)	n/a
Barbuto (2017)	RCT: 4x YN (iRest) in 4 weeks plus CD	60 employees (30 each in EG and CG)	Reduction of stress (PSS, diary)	n/a

Study	Design	Sample	Effects for Yoga Nidra	**Effect sizes**
Ganpat (2020)	RCT: 3 weeks YN	60 students (30 with YN, 30 without)	Increase in Emotional Intelligence	n/a
Moszeik (2016); Moszeik et al. (2020)	RCT: 30 days YN	771 people (341 EG, 430 WCG)	Decreased stress, negative affect, increased well-being, positive affect, mindfulness, quality of sleep	small
Manik & Gartia (2016)	NRCT-AC: 4 weeks YN	120 people (60 YN, 60 breathing exercises)	Reduction in hypertension, pulse, respiratory rate, BMI and anxiety (HARS) for YN	n/a
Wheeler et al. (2018)	NRCT-AC: 1-11x YN in 8-12 weeks	63 veterans (28 in EG, 35 in ACG with acupuncture)	Greater decrease in stress (PSS-10) for YN	n/a
Li et al. (2019)	NRCT-AC: YN during colonoscopy	144 patients divided into 3 groups (YN, music, no treatment)	Less pain and higher satisfaction for music and YN (YN slightly more), no difference in blood pressure	n/a
Borchardt et al. (2012)	RCT-AC: 1x/week YN over one month	75 female students (25 each in YN EG, Progressive Relaxation ACG, audio book ACG)	Largest decrease in cortisol and largest increase in positive affect (cortisol, PANAS) for YN	n/a
Markil et al. (2012)	RCT-AC: 1xYN	20 students (EG: Yoga + YN, ACG: Yoga only)	Effect on heart rate even without yoga	n/a

Study	Design	Sample	Effects for Yoga Nidra	**Effect sizes**
Gutman et al. (2016)	RCT-AC: 14 days YN (iRest)	29 people with sleep problems (9 in EG, 10 in Dreampad Pillow ACG, 10 in Sleep Hygiene ACG)	Longer sleep duration for YN; less waking up for ACG; otherwise, no differences (GSDS, PSQI, sleep monitor, sleep diary)	medium to big
Immink (2016)	RCT-AC: 30 min. YN	12 residents of a yoga center with many years of meditation experience (ACG: light work)	Increased cognitive performance (memory)	big
Wahbeh & Nelson (2019)	RCT-AC: 6 weeks YN (iRest)	29 older adults with depressive symptoms (15 in EG: YN, 14 in ACG: music)	Greater decrease in sleep disturbances for YN	n/a

Notes. YN = Yoga Nidra; EG =experimental group, CG = control group; RCT = randomized controlled trial; NRCT-AC = not randomized controlled trial with active control; RCT-AC = randomized controlled trial with active control; PGWI = Psychological General Wellbeing Index; BMI = body mass index; PSS = Perceived Stress Scale; SSSI = Smith Stress Symptoms Inventory; DVPRS = Defense and Veterans Pain Rating Scale; PGIC = Patient Global Impression of Change; GSDS = General Sleep Disturbance Scale; HARS= Hamilton anxiety rating scale; VAS = visual analog scale for life stress intensity level;

Table S2

Study Procedure

Total *N* = 362	t1(baseline)	Intervention(2 months)	t2	t3
R EG1 (*n* = 101)	Q1 C1	X	O2 C2	O3 C3
R EG2 (*n* = 80)	Q1 C1	X	O2 C2	O3 C3
R AC (*n* = 74)	Q1 C1	X	O2 C2	O3 C3
R WC (*n* = 107)	Q1 C1		O2 C2	O3 C3

Notes. R = Randomization, Q = Online questionnaire, C = Salivary Cortisol Collection (optional), X = Treatment, t1 = baseline/before the intervention, t2 = immediately after the intervention, t3 = 3 months after the intervention

Table S3

Overview of Measures

	t1	t2	t3
Stress Screening	x	x	x
Anxiety	x	x	x
Depression	x	x	x
Rumination	x	x	x
Satisfaction with Life	x	x	x
Sleep quality	x	x	x
Mindfulness	x	x	x
Optional: Stress profile via Salivary Cortisol	x	x	x

Notes. t1 = baseline/before the intervention, t2 = immediately after the intervention, t3 = follow-up 3 months later

Table S4

Distribution of demographic characteristics across the experimental groups

	EG1 *n* = 101 Quantity (%)	EG2 *n* = 80 Quantity (%)	ACG *n* = 74 Quantity (%)	WCG *n* = 107 Quantity (%)
**Age**				
19 – 35 years	43 (44.33%)	29 (36.71%)	28 (39.44%)	37 (41.57%)
36 – 50 years	34 (35.05%)	27 (34.18%)	26 (36.62%)	33 (37.08%)
51 – 65 years	18 (18.56%)	20 (25.31%)	17 (23.94%)	17 (19.10%)
65 – 80 years	2 (2.06%)	3 (3.80%)	-	2 (2.25%)
**Gender**				
Male	18 (18.37%)	11 (13.92%)	17 (23.94%)	18 (20.22%)
Female	79 (80.61%)	68 (86.08%)	54 (76.06%)	71 (79.78%)
Diverse	1 (1.02%)	-	-	-
**Children**				
Yes	42 (42.86%)	38 (48.10%)	26 (36.62%)	47 (52.80%)
No	56 (57.14%)	41 (51.90%)	45 (63.38%)	42 (47.19%)
**Education**				
Lower school	1 (1.03%)	-	1 (1.41%)	3 (3.37%)
Intermediate school	9 (9.28%)	11 (13.92%)	13 (18.31%)	10 (11.24%)
Applied Sciences qual.	11 (11.34%)	9 (11.39%)	5 (7.04%)	8 (8.99%)
University qual.	23 (23.71%)	28 (35.44%)	15 (21.13%)	25 (28.09%)
University	53 (54.64%)	31 (39.24%)	37 (52.11%)	43 (48.31%)
**Leadership position**				
Yes	23 (23.47%)	16 (20.25%)	22 (30.99%)	21 (23.60%)
No	75 (76.53%)	63 (79.75%)	49 (69.01%)	68 (76.40%)
**Sportiness**				
None	3 (3.06%)	5 (6.33%)	3 (4.23%)	5 (5.62%)
Little	30 (30.61%)	21 (26.58%)	20 (28.17%)	17 (19.10%)
Medium	35 (35.72%)	39 (49.37%)	35 (49.29%)	34 (38.20%)
Much	22 (22.45%)	10 (12.66%)	10 (14.08%)	26 (29.21%)
Very much	8 (8.16%)	4 (5.06%)	3 (4.23%)	7 (7.87%)
**Living environment**				
City	33 (57.89%)	36 (69.23%)	23 (53.49%)	49 (63.64%)
Countryside	24 (42.11%)	16 (30.77%)	20 (46.51%)	28 (36.36%)

Notes. t1 = baseline/before the intervention, t2 = immediately after the intervention, t3 = follow-up 3 months later, WCG = waitlist control group, ACG = active control group with music intervention, EG1 = 11 min Yoga Nidra, EG2 = 30 min Yoga Nidra

Table S5

Descriptive Data of the Dependent Variables within the 4 Groups

	t1 *M(SD)*	t2 *M(SD)*	t3 *M(SD)*
Stress	n = 337, α = .91	n = 265, α = .91	n = 229, α = .91
EG1	2.89(0.84)	2.57(0.76)	2.59(0.81)
EG2	2.83(0.73)	2.36(0.64)	2.37(0.56)
ACG	2.79(0.76)	2.54(0.58)	2.56(0.62)
WCG	2.73(0.80)	2.64(0.75)	2.54(0.70)
Anxiety	n = 337, α = .91	n = 265, α = .91	n = 229, α = .90
EG1	2.26(0.69)	2.02(0.64)	2.00(0.64)
EG2	2.11(0.54)	1.92(0.49)	1.90(0.42)
ACG	2.13(0.54)	2.01(0.54)	2.09(0.58)
WCG	2.14(0.64)	2.11(0.65)	2.06(0.57)
Depression	n = 337, α = .92	n = 265, α = .92	n = 229, α = .92
EG1	2.15(0.62)	2.00(0.65)	2.03(0.67)
EG2	2.04(0.56)	1.90(0.47)	1.92(0.42)
ACG	2.02(0.58)	2.06(0.64)	2.07(0.67)
WCG	1.98(0.56)	1.99(0.62)	2.01(0.59)
Rumination	n = 337, α = .91	n = 265, α = .92	n = 229, α = .93
EG1	3.30(0.77)	3.08(0.75)	2.95(0.82)
EG2	3.31(0.72)	3.04(0.77)	2.85(0.82)
ACG	3.19(0.89)	3.09(0.85)	2.94(0.83)
WCG	3.14(0.84)	3.20(0.92)	3.10(0.84)
Satisfaction with life	n = 337, α = .89	n = 265, α = .90	n = 229, α = .91
EG1	4.91(1.24)	5.08(1.26)	5.26(1.21)
EG2	5.00(1.12)	5.19(1.10)	5.20(1.08)
ACG	4.68(1.33)	4.85(1.27)	4.76(1.27)
WCG	5.04(1.12)	4.92(1.10)	4.98(1.15)
Sleep disturbances	n = 335, α = .77	n = 265, α = .78	n = 229, α = .76
EG1	0.85(0.41)	0.77(0.40)	0.73(0.41)
EG2	0.84(0.39)	0.70(0.39)	0.71(0.33)
ACG	0.82(0.43)	0.77(0.40)	0.75(0.44)
WCG	0.73(0.40)	0.74(0.41)	0.71(0.37)

	t1 *M(SD)*	t2 *M(SD)*	t3 *M(SD)*
Observing	n = 337, α = .67	n = 265, α = .73	n = 229, α = .72
EG1	3.51(0.87)	3.62(0.94)	3.61(0.83)
EG2	3.65(0.75)	3.98(0.68)	3.92(0.68)
ACG	3.55(0.83)	3.73(0.85)	3.59(0.94)
WCG	3.45(0.87)	3.56(0.88)	3.61(0.85)
Nonreactivity	n = 337, α = .84	n = 265, α = .87	n = 229, α = .84
EG1	2.84(0.91)	3.22(0.95)	3.15(0.97)
EG2	2.81(0.89)	2.98(1.02)	3.12(0.89)
ACG	2.87(0.93)	3.01(0.86)	3.16(0.78)
WCG	2.87(0.92)	2.95(0.98)	3.03(0.96)
Describing	n = 337, α = .86	n = 265, α = .88	n = 229, α = .88
EG1	3.58(0.96)	3.72(1.00)	3.75(1.01)
EG2	3.84(0.96)	4.02(0.83)	3.90(0.81)
ACG	3.54(1.01)	3.71(0.94)	3.65(0.98)
WCG	3.55(0.93)	3.75(0.96)	3.69(0.97)
Accepting without judging	n = 337, α = .82	n = 265, α = .84	n = 229, α = .81
EG1	3.75(1.01)	3.98(1.01)	4.03(0.98)
EG2	4.02(0.88)	4.19(0.88)	4.24(0.73)
ACG	3.90(0.86)	4.06(0.83)	4.13(0.78)
WCG	4.00(0.91)	4.00(0.91)	4.08(0.84)
Acting with awareness	n = 337, α = .69	n = 265, α = .69	n = 229, α = .74
EG1	3.09(0.82)	3.22(0.77)	3.35(0.91)
EG2	3.27(0.73)	3.57(0.76)	3.46(0.72)
ACG	3.24(0.75)	3.41(0.70)	3.43(0.76)
WCG	3.18(0.82)	3.40(0.84)	3.40(0.81)
Total cortisol	n = 141	n = 127	n = 112
EG1	423.35(115.57)	464.53(88.33)	454.38(77.00)
EG2	459.45(102.79)	452.68(89.44)	451.82(88.33)
ACG	443.25(86.62)	458.56(106.36)	456.60(70.83)
WCG	431.51(97.58)	469.69(101.10)	466.51(99.57)

	t1 *M(SD)*	t2 *M(SD)*	t3 *M(SD)*
Cortisol awakening response	*n* = 135	*n* = 123	*n* = 105
EG1	.305(.32)	.227(.54)	.208(.49)
EG2	.310(.30)	.068(.42)	.219(.54)
ACG	.235(.43)	.204(.38)	.178(.48)
WCG	.190(.40)	.197(.36)	.278(.41)
Cortisol flattening	*n* = 141	*n* = 126	*n* = 114
EG1	-.044(.02)	-.044(.02)	-.046(.02)
EG2	-.042(.01)	-.047(.02)	-.045(.02)
ACG	-.046(.02)	-.049(.02)	-.044(.02)
WCG	-.044(.01)	-.047(.01)	-.044(.02)

Notes. t1 = baseline/before the intervention, t2 = immediately after the intervention, t3 = follow-up 3 months later, WCG = waitlist control group, ACG = active control group with music intervention, EG1 = 11 min Yoga Nidra, EG2 = 30 min Yoga Nidra; the different n of cortisol data are based on missing values.

Table S6

Effects of 11 min Yoga Nidra Compared to Waitlist Control

	Estimate Grand Mean (*SE*)	Estimate Interaction effect (*SE*)	Evidence Interaction effect	*d*	95% CI		*p* in %	Bayes Factor
Stress	2.90 (0.08)	-0.24 (0.09)	-2.61	-0.14	-0.42	-0.06	0.45	219.82
Anxiety	2.16 (0.06)	-0.20 (0.07)	-2.99	-0.16	-0.34	-0.07	0.14	704.95
Depression	2.00 (0.05)	-0.15 (0.06)	-2.5	-0.14	-0.27	-0.03	0.62	160.04
Rumination	3.30 (0.08)	-0.23 (0.08)	-2.8	-0.15	-0.40	-0.07	0.24	414.52
Life satisfaction	4.92 (0.12)	0.17 (0.09)	1.83	0.10	-0.01	0.34	96.66	28.96
Observing	3.46 (0.09)	0.09 (0.11)	0.83	0.04	-0.12	0.30	79.72	3.93
Nonreactivity	2.86 (0.09)	0.26 (0.11)	2.43	0.13	0.05	0.47	99.24	131.43
Describing	3.53 (0.10)	-0.06 (0.10)	-0.62	-0.03	-0.25	0.13	26.81	2.73
Accepting without judging	3.97 (0.09)	0.24 (0.11)	2.22	0.12	0.03	0.44	98.67	74.12
Acting with awareness	3.20 (0.08)	-0.05 (0.11)	-0.43	-0.02	-0.25	0.16	33.41	2.00
Sleep disturbances	0.84 (0.04)	-0.08 (0.05)	-1.60	-0.08	-0.18	0.02	5.52	17.11
Total Cortisol	433.31 (15.19)	2.48 (25.18)	0.10	<0.01	-46.87	51.83	53.92	1.17
Cortisol awakening response	0.19 (0.07)	-0.11 (0.12)	-0.91	-0.03	-0.34	0.12	18.05	4.54
Cortisol flattening	-0.04 (<0.01)	<0.01 (<0.01)	0.75	0.03	<-0.01	0.01	77.34	3.41

Notes. Estimate = Maximum Likelihood Estimate (matches Bayesian estimate under a flat prior), SE = Standard Error (matches Bayesian posterior standard deviation under a flat prior), Evidence = Estimate/SE, d = Cohen's d was calculated as evidence/square root N, CI = confidence interval, p = posterior positive, posterior probability that the population parameter is above zero under a flat prior (consistent with the p-value of a one-tailed likelihood ratio test against the null hypothesis, that the parameter is zero), Bayes factor = ratio of the probabilities under the two competing hypotheses (effect > 0 vs. effect <= 0).

Table S7

Effects of 11 min Yoga Nidra Compared to Active Control Group with Music

	Estimate Grand Mean (*SE*)	Estimate Interaction effect (*SE*)	Evidence Interaction effect	*d*	95% CI		*p* in %	Bayes Factor
Stress	2.90 (0.08)	-0.08 (0.11)	-0.78	-0.04	-0.29	0.13	21.79	3.59
Anxiety	2.13 (0.07)	-0.10 (0.08)	-1.29	-0.07	-0.25	0.05	9.93	9.07
Depression	2.16 (0.06)	-0.18 (0.07)	-2.47	-0.13	-0.33	-0.04	0.67	148.26
Rumination	3.30 (0.08)	-0.10 (0.09)	-1.13	-0.06	-0.27	0.07	13.03	6.67
Life satisfaction	4.92 (0.12)	-0.07 (0.12)	-0.62	-0.03	-0.30	0.16	26.92	2.72
Observing	3.50 (0.09)	-0.05 (0.01)	-0.42	-0.02	-0.27	0.17	33.87	1.95
Nonreactivity	2.86 (0.11)	0.19 (0.13)	1.38	0.07	-0.08	0.45	91.63	10.95
Describing	3.53 (0.11)	0.11 (0.12)	0.88	0.05	-0.13	0.34	81.12	4.30
Accepting without judging	3.91 (0.10)	0.11 (0.13)	0.82	0.04	-0.15	0.37	79.34	3.84
Acting with awareness	3.25 (0.09)	-0.03 (0.01)	-0.23	-0.01	-0.25	0.20	40.90	1.45
Sleep disturbances	0.97 (0.05)	-0.02 (0.06)	-0.37	-0.02	-0.14	0.09	35.46	1.82
Total Cortisol	445.39 (15.86)	25.42 (28.80)	0.88	0.03	-31.03	81.87	81.13	4.30
Cortisol awakening response	0.25 (0.08)	-0.06 (0.12)	-0.47	-0.02	-0.28	0.17	31.91	2.13
Cortisol flattening	-0.05 (0.02)	<0.01 (<0.01)	0.22	0.01	-0.01	0.01	58.60	1.42

Notes. Estimate = Maximum Likelihood Estimate (matches Bayesian estimate under a flat prior), *SE* = Standard Error (matches Bayesian posterior standard deviation under a flat prior), Evidence = Estimate/SE, *d* = Cohen's d was calculated as evidence/square root *N*, *CI* = confidence interval, *p* = posterior positive, posterior probability that the population parameter is above zero under a flat prior (consistent with the p-value of a one-tailed likelihood ratio test against the null hypothesis, that the parameter is zero), Bayes factor = ratio of the probabilities under the two competing hypotheses (effect > 0 vs. effect <= 0).

Table S8

Effects of 30 to 11 min Yoga Nidra (EG2 to EG1)

	Estimate Grand Mean (*SE*)	Estimate Interaction effect (*SE*)	Evidence Interaction effect	*d*	95% CI		*p* in %	Bayes Factor
Stress	2.90 (0.08)	-0.11 (0.11)	-1.08	-0.06	-0.32	0.09	14.09	6.10
Anxiety	2.11 (0.06)	-0.05 (0.09)	-0.58	-0.03	-0.22	0.12	28.21	2.50
Depression	2.03 (0.07)	-0.03 (0.08)	-0.36	-0.02	-0.19	0.13	35.72	1.80
Rumination	3.30 (0.08)	-0.07 (0.10)	-0.73	-0.04	-0.26	0.12	23.21	3.31
Life satisfaction	4.92 (0.12)	0.01 (0.12)	0.09	<0.01	-0.23	0.25	53.56	1.15
Observing	3.65 (0.09)	0.17 (0.12)	1.42	0.08	-0.06	0.40	92.20	11.82
Nonreactivity	2.83 (0.09)	-0.18 (0.14)	-1.30	-0.07	-0.44	0.09	9.65	9.36
Describing	3.84 (0.10)	0.03 (0.10)	0.32	0.02	-0.17	0.23	62.69	1.68
Accepting without judging	4.02 (0.10)	0.10 (0.12)	0.79	0.04	-0.14	0.34	78.48	3.65
Acting with awareness	3.08 (0.08)	0.22 (0.12)	1.88	0.10	-0.01	0.44	96.98	32.14
Sleep disturbances	0.94 (0.05)	-0.05 (0.06)	-0.80	-0.04	-0.16	0.07	21.28	3.70
Total Cortisol	423.90 (18.22)	-43.47 (27.62)	-1.57	-0.06	-97.61	10.67	5.78	16.31
Cortisol awakening response	0.30 (0.05)	-0.15 (0.12)	-1.19	-0.04	-0.38	0.09	11.73	7.52
Cortisol flattening	-0.04 (<0.01)	-0.01 (0.01)	-1	-0.04	-0.01	<0.01	15.87	5.30

Notes. Estimate = Maximum Likelihood Estimate (matches Bayesian estimate under a flat prior), *SE* = Standard Error (matches Bayesian posterior standard deviation under a flat prior), Evidence = Estimate/SE, *d* = Cohen's d was calculated as evidence/square root *N*, *CI* = confidence interval, *p* = posterior positive, posterior probability that the population parameter is above zero under a flat prior (consistent with the p-value of a one-

tailed likelihood ratio test against the null hypothesis, that the parameter is zero), Bayes factor = ratio of the probabilities under the two competing hypotheses (effect > 0 vs. effect <= 0).

Table S9

Frequency in all 3 Intervention Groups (t2)

	ACG<br>*n = 51*	*EG1*<br>*n =62*	*EG2*<br>*n = 62*
All 57 days	1 (2%)	6 (9.7%)	0
39-56 days	17 (33.3%)	16 (25.8%)	19 (30.6%)
21-38 days	12 (23.5%)	17 (27.4%)	10 (16.1%)
9-20 days	14 (27.5%)	13 (21%)	17 (27.4%)
2-8 days	5 (9.8%)	7 (11.3%)	12 (19.4%)
1 time	1 (2%)	3 (4.8%)	1 (1.6%)
Not at all	1 (2%)	0	3 (4.8%)

Notes. ACG = active control group with 10 min music intervention, EG1 = 11 min of Yoga Nidra, EG2 = 30 min of Yoga Nidra

Table S10

Further exploratory results

	ACG	*EG1*	*EG2*
Gender	Men had a steeper rise in total cortisol than women.	Men showed a greater effect on a flatter morning cortisol rise and a lower increase in observing.	-
Age	Older participants experienced greater relaxation in the evening, with a steeper cortisol flattening and higher scores on acceptance without judging.	Older individuals reported lower stress and higher scores on the describing facet.	Older participants showed higher morning cortisol activation.
Leadership position	-	Holding a leadership position was correlated with a greater decrease in sleep disturbances and total cortisol concentration.	-
Shiftwork	-	-	-
Education	Higher education was associated with greater evening relaxation, steeper cortisol flattening, and higher scores on observing.	-	-
Children	-	Participants with children reported a higher decrease in stress.	-

	ACG	EG1	EG2
Sportiness	-	More sporty individuals showed a higher increase in total cortisol.	-
Living Environment	Individuals living in the countryside had a significantly flatter cortisol awakening response compared to those living in urban areas.	-	-

Notes. ACG = active control group with 10 min music intervention, EG1 = 11 min of Yoga Nidra, EG2 = 30 min of Yoga Nidra

Figure S1

SEM as an Extended ANOVA Design to Analyze the Data with Full Information Maximum Likelihood (FIML).

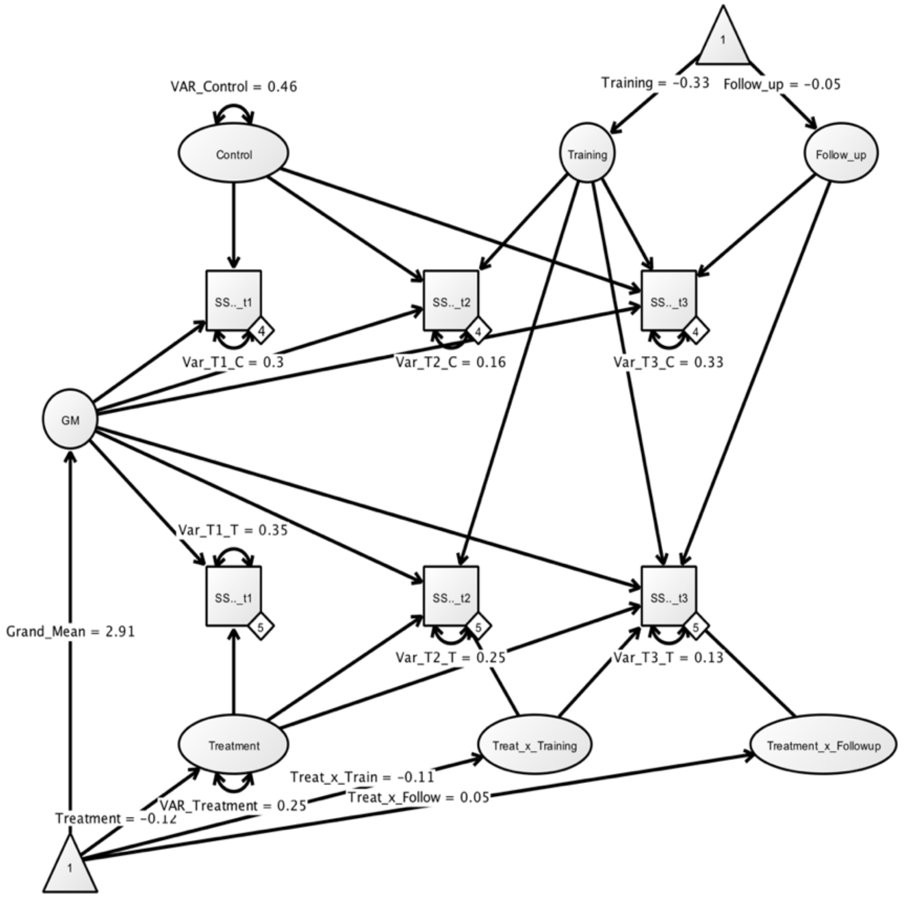

Note. The latent variables "treatment", "training" and "follow-up" describe the main effects; "Treat x Training" and "Treatment x Follow-up" describe the interaction effects. The variable "Treatment" has a residual variance to model the common variance of the intervention group. An analogous variable "Control" provides the common variance

of the comparison group; This variable has no mean, so the mean of the intervention provides the additional effect of the intervention in addition to the grand mean represented in the latent variable “GM”. All residuals between groups and measurement times may be different.

Figure S2

Consort Flow Chart of all Participants of the Study

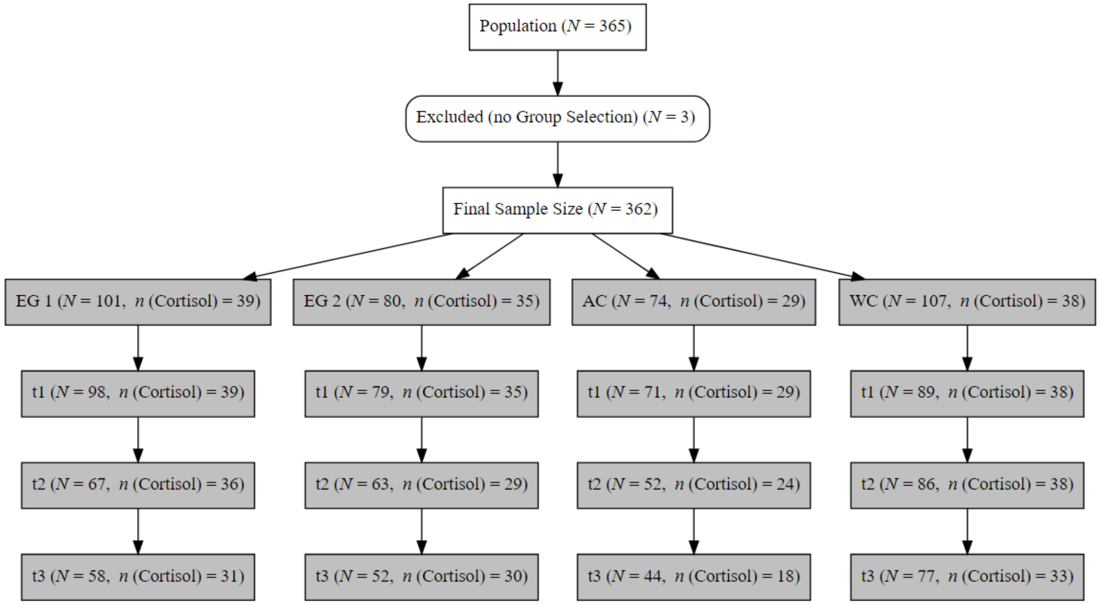

Note. t1 = baseline/before the intervention, t2 = immediately after the two months intervention, t3 = three months after the intervention, EG1 = 11 min of Yoga Nidra, EG2 = 30 min of Yoga Nidra, AC = active control group with 10 min music, WC = waiting control group

Figure S3

Changes of Diurnal Cortisol Values from t1 to t3.

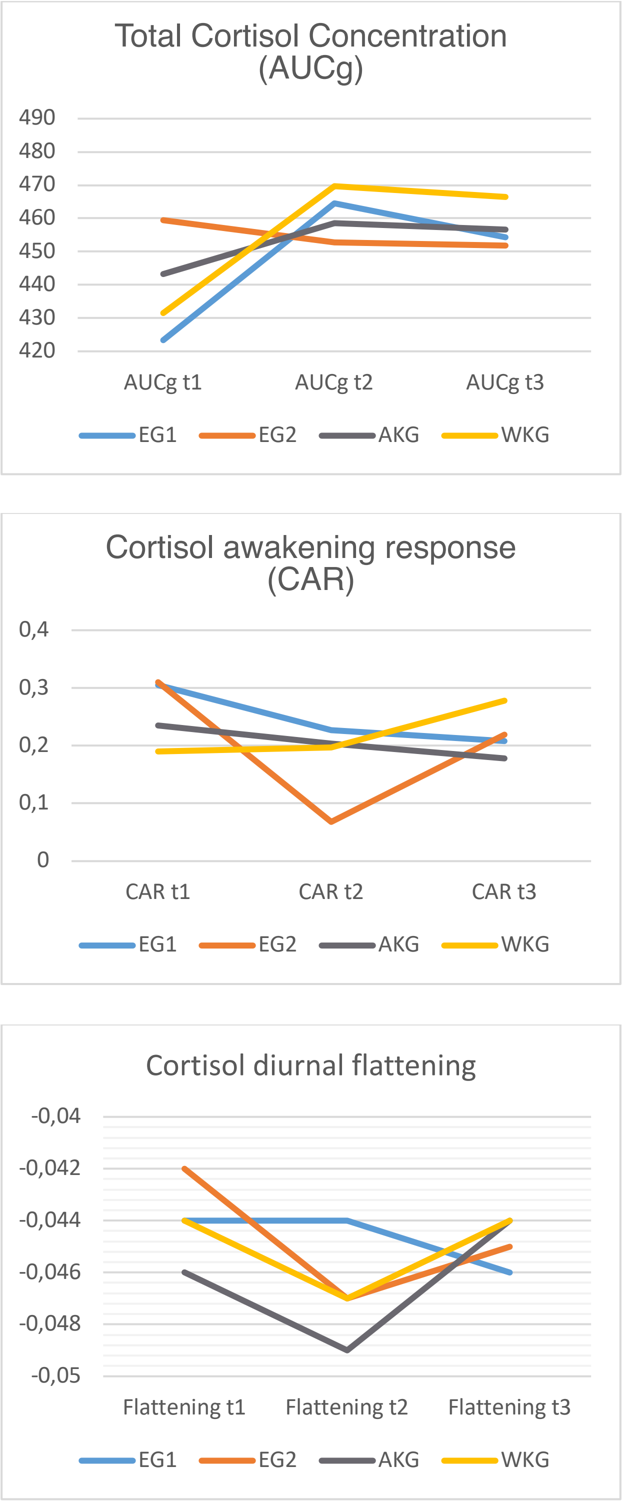

Note. t1 = baseline/before the intervention, t2 = immediately after the two months intervention, t3 = three months after the intervention, EG1 = 11 min of Yoga Nidra, EG2 = 30 min of Yoga Nidra, AKG = active

control group with 10 min music, WKG = waiting control group; n = 229; AUCg = nmol/l*min, CAR and flattening = nmol/l*h (log-transformed)
